# Applying the economic concept of profitability to leaves

**DOI:** 10.1038/s41598-020-79709-w

**Published:** 2021-01-08

**Authors:** Rafael Villar, Manuel Olmo, Pedro Atienza, Antonio J. Garzón, Ian J. Wright, Hendrik Poorter, Luis A. Hierro

**Affiliations:** 1 Area de Ecología, Dpto de Botánica, Ecología y Fisiología Vegetal, Facultad de Ciencias, Universidad de Cordoba, Cordoba, Spain; 2 Departamento de Economía e Historia Económica, Universidad de Sevilla, Sevilla, Spain; 3 Department of Biological Sciences, Macquarie University, North Ryde, NSW 2109 Australia; 4 Plant Sciences (IBG-2), Forschungszentrum Jülich GmbH, 52425 Jülich, Germany

**Keywords:** Ecology, Plant sciences

## Abstract

Economic principles can be extended to biological organisms as they optimize the use of resources, but their use in biology has been limited. We applied concepts from traditional economics to the main production unit of plants, the leaf. We quantified the profitability (profit/cost of investment) of leaves from seven biomes worldwide and compared those to the profitability of companies. Here we demonstrate for the first time key similarities and differences between leaf and human economics. First, there was a weak, but positive relationship between profitability and size, both for leaves and companies. Second, environment has a strong effect on profitability, with high values in leaves from biomes with short growth periods and, for companies associated with innovation. Third, shorter longevity of productive units was related to higher profitability. In summary, by comparing economic behaviours of plants and humans there is potential to develop new perspectives on plant ecological strategies and plant evolution.

## Introduction

There are various definitions of economy, which generally revolve around three basic concepts: wealth, exchange and scarcity. Originally, classical economists defined the economy according to the object of study: wealth, its origin^1^, its growth^2^ and its distribution^3^. Most classical economists proposed free trade as the ideal system for achieving greater wealth and a more adequate distribution of it. This gives rise to the second concept that has been used for the definition of economy, exchange. According to Boulding^59^ “*The world of economics is organized fundamentally by exchange. This relationship, by which each of two parties gives something to the other and receives something in return, is indeed a powerful social organizer*”. Finally, the third concept on which the definitions of economics have been built is scarcity. In this perspective, economics is “…*the science which studies human behaviour as a relationship between ends and scarce means which have alternative uses*”^4^.

So, to what extent can these concepts be applied fruitfully to plants, to understand their evolution and behaviours? A plant acquires its main resources (water, nutrients, light, CO_2_) in order to ensure growth, survival and ultimately, reproduction. There have been attempts to understand the economic nature of plant functioning. Bloom et al.^5^ introduced the idea to consider the carbon budget of a leaf or plant from an economic perspective, analysing the commonalities between plants and companies from a theoretical perspective. But so far, there have been few attempts to take this approach further, despite the widespread use of economic analogies in plant ecology^6–14^. In this work we revisit this issue, focusing on scarcity (and the related concept of profitability)—reporting a comparison of leaf and company economic features, based on a global leaf dataset, and company data from Spain.

In our view it is the *scarcity* element that best allows the possibility of assimilating certain plant activities to economic behaviour. Robbins' concept cited above proposes that we talk of economics when we refer to human behaviours guided by an optimizing goal in the use of scarce resources. Our objective is to demonstrate the feasibility of applying economic concepts to biological processes of plants, not only from a theoretical point of view, as has already been proposed^5,15^, but from an empirical point of view. For this we focus on one of the most fundamental economic parameters, profitability. In economics, the relevance of profitability is especially clear. In essence, all economic behaviour is based on the maximization of profit and therefore on profitability^1^, which is nothing other than relative benefit. It is assumed that any business decision is guided by the objective of maximizing profitability: investments, changes in the organization of production, actions aimed at reducing costs or increasing revenue, etc.

**Figure 1:**
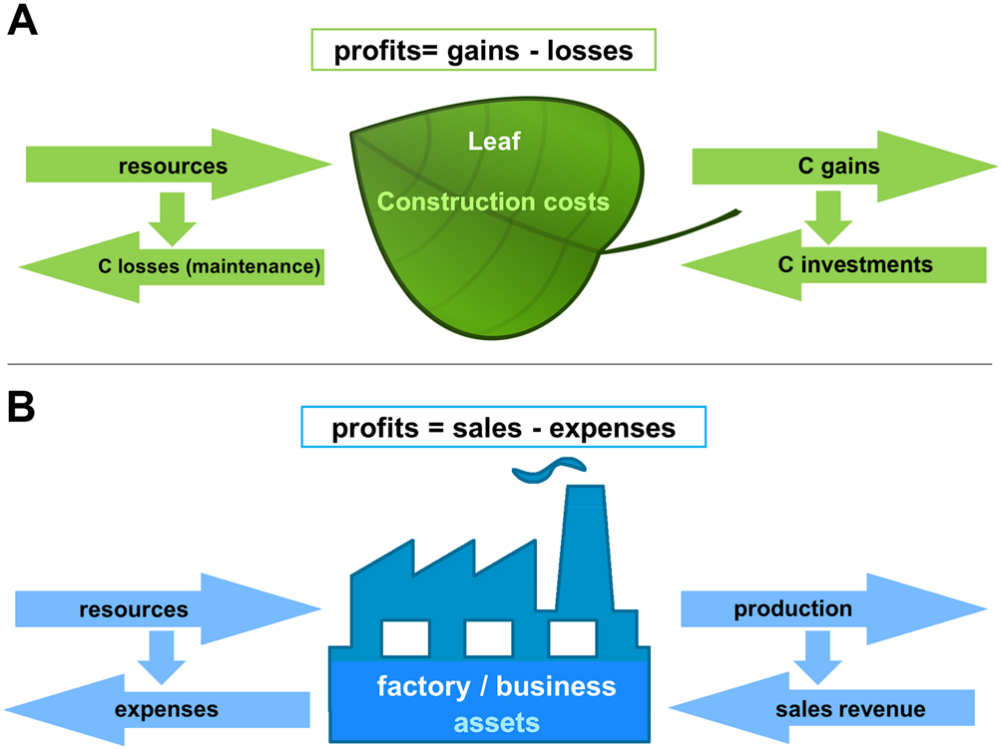
Scheme of general economic functioning of leaves and companies. (**A**) Leaves show similar behaviour to companies. A leaf acquires different resources (CO_2_, light, nutrients and water) to synthesize carbohydrates (C gains) which can be used for maintenance (C expenses) or stored and/or exported to be used in various ways (transport of compounds to other organs, construction of new organs, etc.) (C investments). The carbon profit of the leaf will be then the difference between the carbon gains and the carbon expenses. (**B**) A company acquires the resources (work, capital and natural resources) through financial investment in activities (expenses) to make different products that are sold, resulting in gains (sales revenue). The overall financial profit of these activities will be the difference between the sales and the expenses.

In this work we explore the analogies that exist between companies and the simplest productive unit of plants, the leaf, based on similarities in their structure and function^5^. The company can be analysed from multiple perspectives. Bloom et al.^5^ analysed the analogy plant/company, but considering the perspective of scarcity, the leaf/company analogy is also valid (see Supplementary File S1 online for a detailed explanation). Under this perspective, companies (or leaves) present themselves as organizing entities of production, using the available resources efficiently for production with the objective to obtain maximum production at minimum cost, maximizing profit and, consequently, the return on capital invested in it. We must also point out that the use of the leaf/company analogy for empirical analysis is the most feasible one. The empirical study of the plant/company analogy in the manner of Bloom et al.^5^ is difficult because there are so few plant datasets with the necessary whole-plant information. Fortunately, the versatility of the company concept makes possible the analysis of the leaf/company analogy since there are extensive databases for both objects of study. We have relatively many leaf-level data on carbon investments, gains and expenses across their lifetime based on the global data set of *leaf economics spectrum* (LES)^13^. For company data we rely on the ‘Annual Iberian Balance Sheet System’ (SABI) database for Spanish companies. In summary, as production units there is an analogy between leaves and companies (Fig. 1, Supplementary Table S1 online).

A company acquires the resources (work, capital and natural resources, including the payments for obtaining resources and the expenses for assets, such as machinery) through financial investment in activities (expenses) to make different products that are sold obtaining financial gains (sales revenue). The profit of these activities will be the difference between the sales and the expenses. Similarly, a leaf acquires different resources (CO_2_, light, nutrients and water) to synthesize carbohydrates (C gains) which can be used for maintenance (C expenses) or stored and/or used in various ways (transport of compounds, construction of new organs, etc.)^16^. The carbon profit of the leaf will then be the difference between the carbon gains and the carbon expenses. At the very least, leaves need to assimilate enough carbon during their life time to payback their own investment in construction^15,17,18^; and, presumably more than that, payback some proportion of the construction and maintenance costs of stems and roots^19^. Further profits are required to ensure positive plant growth, for defence, feeding root symbionts (which aid in nutrient uptake), and reproduction.

For our analysis we determined the economic profitability of leaves, which is the percentage of investment in leaf construction obtained as surpluses that can be used to manufacture other leaves or other plant organs, whether to maintain plant metabolism or to invest in reproduction. In short, it is the profit/cost ratio of investment. We know of only one study that uses the term “leaf profitability”^20^, and after that the term has not been used. Several studies^15,21–23^ have evaluated the leaf payback time or the minimum leaf longevity, which is the time that a leaf requires to amortize its construction costs, calculated as the leaf construction cost divided by the leaf’s average net daily carbon fixation. Thus, the payback time is similar to the inverse of profitability.

We have used published leaf data for a broad range of species (630 species) covering a wide range of the *leaf economic spectrum* (LES^13^). Wright et al.^13^ found that leaves with high leaf mass per area (LMA, leaf dry mass per unit leaf area) have a lower photosynthetic rate per unit leaf mass, but longer leaf lifespans^13,15^. We calculated leaf benefits (photosynthesis) and expenses (maintenance respiration) across the leaf life time. Then, the leaf profitability was calculated as the ratio of profit to cost of investment**.** Next, we show the estimates of leaf profitability in relation to leaf size, different environments (biomes) and leaf durability and compare these patterns with those relative to companies. The comparison with companies is carried out with the purpose to see how the concept of profitability behaves in the economic reality of the leaf and to verify that there are similarities that allow us to shed light on a future potentiality of this concept.

We consider that the fundamental advantage of studying leaf profitability with respect to other metrics (payback time, lifetime C balance of a leaf, etc.) is that profitability is a basic concept in economy. The profitability that we have calculated is an economic concept applied to a biological process, which allows us to determine a surplus of carbon produced, and which is expressed in relative terms and therefore suitable for comparison. Nonetheless, we note that this is an initial step; in principle the analysis could be extended to other parts of the plant, to a plant as a whole, or to each species of a biome or geographic area. The calculation of a relative magnitude such as profitability provides a pathway to asking new research questions, for example: If profitability is related to biological success or survival, how will environmental changes such as increasing temperatures and atmospheric CO_2_ concentration, or altered growing season, affect leaf-level profitability? How much will this vary between species with different growth forms or phenologies? What are the implications for vegetation composition and biome boundaries? Introducing an economic approach can provide answers to questions relevant to biology and allows us to capture part of the methodological arsenal developed by the economy. Obviously, if we want to apply economic principles to plants it is necessary to calculate the variables that economic models use. This is a first step to identify variables associated with economic behaviour in plants, facilitating the application of economic models to plants in future studies.

In summary, the main objective of this study is to know if the concept of profitability can be applied to the economic reality of the leaf and to verify that there are similarities with companies that allow us to demonstrate future potential for this approach. For that, we calculated the economic profitability of leaves and companies and analysed the patterns in relation to size, environment and durability (longevity, lifespan). We hypothesised that the size of a production unit can influence profitability. For example, a larger size may imply a higher cost associated with structure; therefore, the changes in profitability will depend on how gains and expenses vary with size. We also expect that the environment may also have a strong influence on the gains, expenses and profits of leaves. We also seek to unravel the effect of durability on profitability. It is expected that the longevity of the systems could have an influence on the economic profitability. Finally, we would like to know which factors can explain the differences in economic profitability, for example if gains, expenses or assets are related to profitability.

## Material and methods

### Leaf database and leaf profitability calculations

We have used published leaf-level data for a broad range of species covering the *leaf economic spectrum* (*LES*)^13^. We focus on four functional leaf traits: (1) leaf dry mass per area (LMA); (2) photosynthetic rate per leaf area measured under optimal conditions; (3) respiration rate per leaf area; and (4) leaf longevity (LL). Of the 2548 species cases in the *LES*, only 274 had available data for all four functional traits. The variable with the lowest number of data was the respiration rate. To increase the number of cases, we relied on the overall relationship observed between leaf respiration and leaf N concentration^13^. Respiration rate per mass was calculated using the following regression equation: *Respiration*_*mass*_ (nmol CO_2_ g^−1^ s^−1^) = 5.43092 + 1.86629 × [*N*_*mass*_ (%)]^2^ (R^2^ = 0.65; *P* < 0.0001) obtained for data of *LES* where respiration rates and leaf N concentration was measured. Then, respiration per leaf area was calculated by multiplying respiration rate per mass and LMA. The final database obtained in this way contains 630 species-cases. Leaf size of the majority of the species was taken from a recently published global dataset^24^. Here the species were grouped into seven biomes (alpine, grassland, temperate forest, tropical seasonal forest, tropical rain forest, tundra, and woodland). The boreal biome was not considered because of the low number of data (only 3). Woody species were classified according to leaf phenology (deciduous or evergreen).

Leaf benefits (photosynthesis) and expenses (maintenance respiration) during their life time can be calculated very simply or using more complex calculations, where additional assumptions need to be made (see Supplementary File S2 online), with the effect of deriving more realistic values of leaf profitability. We present the results following the more complex approach. In these calculations we considered the changes in benefit and expense rates depending on the leaf age or changes in environmental conditions^25^. Then, the profitability was calculated as the Internal Rate of Return (IRR) (calculated with the formula of IRR in Excel; see Supplementary File S3 online), defined as the ratio of profit to cost of investment, considering the profit as the difference between life-time photosynthetic gains and life-time maintenance respiration expenses; and the cost of investment as the leaf construction costs. We calculated leaf profitability following several different approaches (different age-related changes in physiological rates or using maximum rates; Supplementary File S2 online) and we found the conclusions were very similar (Supplementary File S4 online). Falster et al.^26^ also found that the general patterns for lifetime return of C per unit C invested obtained from simple trait measurements are reliable and commensurate with those obtained from more detailed modelling of lifetime revenue.

The rate of photosynthesis (CO_2_-fixation) and respiration (CO_2_-efflux) were converted to C equivalents and integrated over 24 h to provide the daily C-gain and C-expense of the leaves, as we explain next. We calculated the C gain (photosynthesis) and C expenses (respiration) over the lifetime of the leaf, considering a linear decline of leaf photosynthesis and respiration rates with leaf age^15,19^ and the growing season and daylight hours for the different biome types (similar to^17^). To calculate the C gain and C expenses of leaves, we need to know the duration of photosynthesis within a year that depends of two components: (a) the duration within a day or mean labor time (***m***), and (b) the duration of the seasonal period favourable for growth (***f***)^19^. We estimate both components for the different biome types (Supplementary File S2 online). We defined the favorable period as being the set of consecutive months that satisfied the following conditions: (1) monthly mean 24-h temperature ≥ 5 °C; and (2) monthly rainfall/evapotranspiration ≥ 0.05, following^24^. To calculate monthly mean rainfall, temperature and evapotranspiration of each biome we selected different coordinates (at least 10) along the geographical range of each biome. Climatic data were obtained using the geovisualization tool for broadcasting climatic data *Global Climate Monitor* (http://www.globalclimatemonitor.org), which is based on the CRU TS3.21 version of the Climate Research Unit (University of East Anglia) database for 1951–2012. In general, favorable period varies strongly with latitude with values of around 90 days for tundra to 364 days for tropical rain forest (Supplementary File S2 online, Supplementary Table S1 online). The favorable and non-favorable period was calculated as the sum of days of favorable and non-favorable days, respectively.

The C gains (photosynthesis) and C expenses (respiration) accumulated across the leaf life-span were calculated following the Eqs. (1 and 2). Kikuzawa^15^ described a linear decline of leaf photosynthesis and respiration rates with leaf age. To satisfy this assumption, averages of photosynthesis and respiration rate were calculated considering it was 50% of the maximum rate. Alternative assumptions of decrease in photosynthetic rate or respiration rate with leaf age did not change the main conclusions (see Supplementary File S4 online)

1 $$Photosynthesis_{{\_{Integrated}}} ({\text{g}}\;{\text{C}}\;{\text{m}}^{ - 2} ) = A\left( {{\text{g}}\;{\text{C}}\;{\text{m}}^{ - 2} \;{\text{h}}^{ - 1} } \right) \times m\left( {{\text{h}}\;{\text{day}}^{ - 1} } \right) \times f\left( {{\text{day}}} \right)$$

2 $$Respiration_{{\_}Integrated} \left( {{\text{g}}\;{\text{C}}\;{\text{m}}^{ - 2} } \right) = R\left( {{\text{g}}\;{\text{C}}\;{\text{m}}^{ - 2} \;{\text{h}}^{ - 1} } \right) \times mr\left( {{\text{h}}\;{\text{day}}^{ - 1} } \right) \times LL$$

*A* and *R* were the rates of photosynthesis and respiration considering 50% of the maximum value to account for the declining of the rate with the leaf age, *m* is the duration of the time favorable for photosynthesis within a day or mean labor time, and *f* is the duration of favorable period. The number of hours per day for respiration (*mr*) was calculated as in Supplementary File S2 online.

The profit accumulated through whole the life of a typical leaf was calculated as the lifetime photosynthetic gains minus lifetime respiration expenses (Eq. 3). The mean daily profit was calculated as the total profit accumulated divided by the leaf longevity (Eq. 4)

3 $$Profit_{{\_}Integrated} ({\text{g}}\;{\text{C}}\;{\text{m}}^{ - 2} ) = Photosynthesis_{{{\_}}Integrated} ({\text{g}}\;{\text{C}}\;{\text{m}}^{ - 2} ){-}Respiration_{{{\_}}Integrated} ({\text{g}}\;{\text{C}}\;{\text{m}}^{ - 2} )$$

4 $$Daily\;profit\;({\text{g}}\;{\text{C}}\;{\text{m}}^{ - 2} \;{\text{day}}^{ - 1} ) = Profit_{{{\_}}Integrated} ({\text{g}}\;{\text{C}}\;{\text{m}}^{ - 2} ){/}leaf\;longevity\left( {{\text{day}}} \right)$$

The internal rate of return (IRR) or profitability of the leaf was calculated with the formula of IRR in Excel (see Supplementary File S3 online) but it can be simplified in many cases as 100 × daily profit/[C investment in the construction of the leaf per unit leaf area (CC_a_)]. The construction cost per unit leaf area is based on the inversion of leaf dry mass per unit leaf area (leaf mass per area, LMA) and the chemical composition of the leaf^27^. Leaf construction cost was calculated based on its known, solid relationship with LMA^18^. Leaf CC_a_ (g glucose m^−2^) was calculated as − 8.0105 + 1.6035 × LMA (R^2^ = 0.97; *P* < 0.001). Leaf CC_a_ was converted to g C m^−2^ by multiplying the g glucose by 0.40 (72 g C in 180 g glucose).

The leaf profitability or internal rate of return (IRR) and the payback time to recover the C investment in the leaf were calculated as:

5 $$Profitability\;(\% {\text{ day}}^{ - 1} ) = 100 \times daily\;profit\;({\text{g}}\;{\text{C}}\;{\text{m}}^{ - 2} \;{\text{day}}^{ - 1} ){/}CC_{a} \;({\text{g}}\;{\text{C}}\;{\text{m}}^{ - 2} )$$

6 $$Payback\;time\left( {{\text{day}}} \right) = CC_{a} \;({\text{g}}\;{\text{C}}\;{\text{m}}^{ - 2} ){/}Daily\;profit\;({\text{g}}\;{\text{C}}\;{\text{m}}^{ - 2} \;{\text{day}}^{ - 1} )$$

To see if our results were affected by our assumptions (specifically, the decline of leaf photosynthetic and respiration rates with leaf age) we calculated the mean daily profit in different ways (Supplementary File S2 online). We found that these assumptions did not affect the main conclusions (see Supplementary File S4 online).

We calculated the economic profitability of leaves and analysed the patterns in relation to leaf size (one-sided surface area), environment (biomes) and durability (longevity). We analysed the relationships between variables with linear regression after log transforming the data. We compared the leaf profitability between biomes and leaf habits (deciduous and evergreens) using one way analysis of variance. The statistical analyses were performed using Statistica (v 8.0^28^).

### Companies’ database and profitability calculations

Data for companies were obtained from the ‘*Sistema Anual de Balances Ibéricos*’ (SABI) database (https://sabi.bvdinfo.com/version-2018119/Login.serv?Code=InvalidIpAddress&LoginParamsCleared=True&LoginResult=nc&product=sabineo&RequestPath=home.serv?product=SabiNeo). The SABI database includes information on book records, descriptive information on the company such as size, ownership structure, and financial variables (sales revenue, expenses, assets, etc.) of 14 years (from 2002 to 2015). We specifically focused on three variables: (1) sales revenue, (2) profits (EBIT: Earnings Before Interest and Taxes), and (3) assets. The annual expenses were calculated as the difference between sales revenue and profits. We use this specific database so as not to mix companies from different countries with different accounting systems, as that may affect the results. From this database we selected the active Spanish companies with data for all the years of the series (88,100 companies). From this population we extracted a random sample of 7000 companies (the maximum allowable data extraction from this database). Then, we calculated the mean values of three variables for the 2002–2015 period: (1) sales revenue, (2) expenses, and (3) assets. Following the definitions of SABI, the annual profit or result of exploitation was calculated as the difference between mean sales revenue and mean expenses per year. We also use data of NASDAQ and Dow Jones companies to compare companies with different longevity.

The economic profitability (IRR) was calculated (see Supplementary File S3 online) with the formula of Internal Rate of Return (IRR) in Excel but it can be simplified in many cases as 100 × annual profit/assets (Eq. 7)

7 $$Economic\;profitability\left( {\% \;{\text{year}}^{ - 1} } \right) = 100 \times result\;of\;exploitation\left( {EUR\;{\text{year}}^{ - 1} } \right)/total\;assets\left( {EUR} \right)$$

Subsequently, we eliminated companies with negative economic profitability and companies with extreme values as identified by a procedure in the SPSS Statistics program (v.24). The extreme values (VE) are those that meet the following condition: Q_1_—3R_Q_ > VE > Q_3_ + 3R_Q_, Q_1_ and Q_3_ being the quartiles 1 and 3, respectively, of the distribution of a sample, and R_Q_ being the interquartile range (R_Q_ = Q_3_—Q_1_). From the initial sample of 7000 companies, 2200 companies were eliminated since part of the sample years included the global economic crisis, during which many companies showed losses and, therefore, negative economic profitability.

The statistical analyses were done for all the companies, and also separately for those with assets lower than 100 × 10^6^ €, as these comprise the majority of companies (99.7%) and to avoid bias from considering the bigger companies. In any case, the results were very similar.

For the comparison between NASDAQ and Dow Jones companies in relation to different longevity we roughly estimated the profitability of the two types of companies by calculating the average growth rate of the value of each of the stock indices for the period 1971–2015 in real terms (i.e. by deducting the growth of prices every year). For that we log-transform the data and calculate the slope of the relationship of Log (stock indices) versus time, giving values of relative growth rate (Supplementary File S6 online).

We analysed the relationships between variables by linear regression after log transforming the data. We compared the profitability of companies of (a) different economic sectors and (b) being NASDAQ or Dow Jones, using one factor analysis of variance. The statistical analyses were performed using Statistica (v 8.0^28^) and IBM SPSS Statistics (v.24).

## Results and discussion

### Leaf profitability

We calculated the economic profitability of leaves and analysed the patterns in relation to leaf size, environment and durability (longevity). There was wide variation in leaf profitability with a mean value (± SD) of 3.4 ± 3.5% day^−1^ and 5th/95th percentile range of 0.29–10.3% day^−1^. There are no previous studies reporting leaf profitability values for direct comparison, but they can be re-calculated based on published values for leaf payback time. For example, Williams et al.^21^ reported payback time values in several *Piper* species from a Mexican rainforest, corresponding to leaf profitability values between 0.01 and 33% day^−1^. Poorter et al.^23^ calculated payback times corresponding to leaf profitability values of 1.25–50% day^−1^ , depending on species type, light environment, and growth conditions (very high values were observed for seedlings grown under non-limiting, hydroponic conditions). The mean values of our study are lower, but in line with values calculated from payback time of Kikuzawa and Lechowicz^25^ (2.2 ± 2% day^−1^), because they consider the mean labour time and the favourable period length, as we also applied in our calculations (see “Methods” and Supplementary File S2 online).

**Figure 2:**
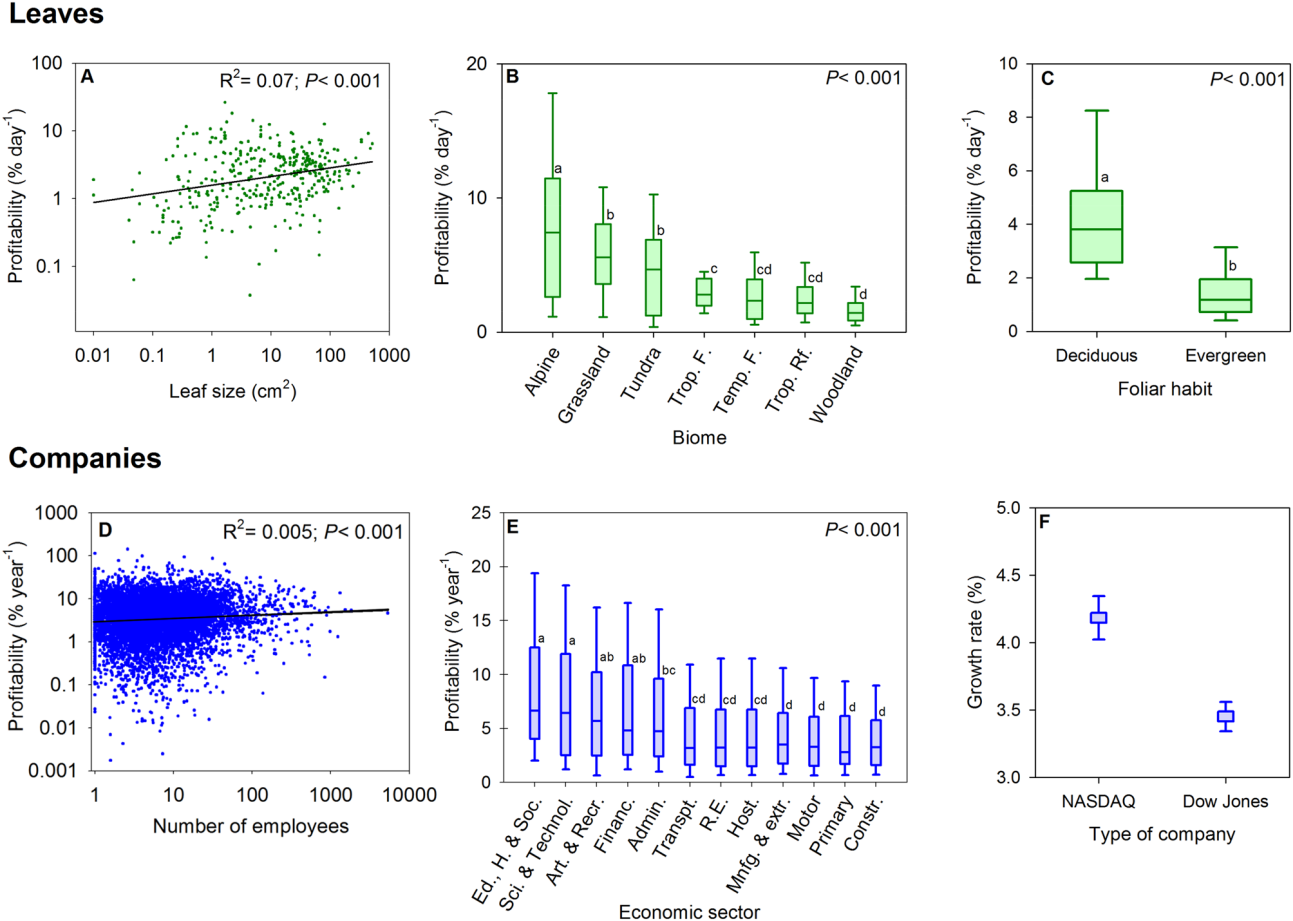
Profitability of leaves and companies in relation to size, environment and durability. For leaves, (**A**) relationships of profitability and leaf size, (**B**) comparison of leaf profitability in different biomes and (**C**) leaf profitability of two different leaf habits (deciduous, short leaf life-span and evergreen, long leaf life-span). Biomes: Alpine, Grasslands, Tundra, Tropical seasonal forest (Trop. F.), Temperate forest (Temp. F.), Tropical rain forest (Trop. RF.), Woodland. For companies, (**D**) relationships of profitability and company size (number of employees), (**E**) comparison of profitability of the different economic sectors (see codes below) and (**F**) average growth rates (similar to profitability) between different types of companies according to technology: NASDAQ companies (technology companies, short-term longevity); and Dow Jones companies (traditional companies, high longevity). Growth rates were calculated as the slope of the trend of market value (see Supplementary File S6 File). Economic sectors: Education, health and social services (Ed., H. & Soc.), Professional, scientific and technical activities (Sci. & Technol.), Artistic, recreational activities and other services (Art. & Recr.), Information and communication and financial and insurance activities (Financ.), Administrative activities and auxiliary services (Admin.), Transportation and storage (Transpt.), Real estate activities (R.E.), Hostelry (Host.), Manufacturing, extractive industries and energy and water supply (Mnfg. & extr.), Retail and wholesale trade and repair of motor vehicles (Motor), Primary (Primary), Construction (Constr.). The box in each plot shows the median and the lower and upper quartile, and the whiskers show the 10 and 90th percentiles.

One factor that could influence profitability is the size of a production unit. For example, a large leaf may imply a higher cost required for structural support; therefore, the changes in profitability will depend on how gains and expenses vary with size. As it turned out, we found that leaf profitability was positively related to leaf size (Fig. 2A), but the percentage of variance explained was not especially high (R^2^ = 0.07, *P* < 0.001).

Higher benefits have been suggested for species with large leaves, for example species with larger leaves tend to deploy branches with a higher ratio of leaf area to stem dry mass^29^, which itself could be associated with a higher growth rate^24,30^ and therefore profitability. However, there are also disadvantages for species with large leaves, for example, higher within-leaf support costs reflected in their higher leaf mass per area (LMA)^29,31,32^, and more structural and less metabolic tissue^32,33^, or an increased risk of overheating or frost damage^24^. Considering global data of many species, Diaz et al.^34^ did not find a strong relationship between leaf size and either LMA or leaf N concentration, both of which are related to photosynthetic gain^13,34^, suggesting that there is no general advantage for a bigger leaf size (but see^35^). Here we assessed the issue more directly, concluding that a meaningful benefit is evident.

The environment may also have a strong influence on the gains, expenses and profits of leaves^16^. We found that leaf profitability varied among biomes (which differ in environmental conditions), with maximum values in the alpine and grasslands biomes and minimum in the woodland biome (Fig. 2B). The high profitability of alpine and grasslands could be related to the short period the leaf is functionally active, thus leaves must be highly profitable to payback the construction cost. Also, most species from alpine and grasslands are herbs and grasses with high photosynthetic rates and low LMA^13,36^, and this may explain the higher profitability. In the case of the woodlands and tropical rain forest the main cause of their low leaf profitability could be the more constant environment that favors evergreen species^15^ which generally have higher LMA and lower photosynthetic rates^13,36^.

We also wanted to unravel the effect of durability on profitability. It is expected that the longevity of the systems could have an influence on the economic profitability. We expect that entities with short longevity should have a higher economic profitability. For example, leaves with a short longevity (e.g. deciduous leaves) have high photosynthetic gains per unit of leaf dry mass and low LMA^13,36,37^ together with a low construction cost per leaf area^18,27^ that could result in higher profitability compared with evergreens. Indeed, we found that leaf profitability was significantly related to the leaf durability, with higher profitability for deciduous compared with evergreen leaves (Fig. 2C). Eamus^38^ also found a lower payback time for deciduous compared with evergreens, which indicates a higher profitability of the deciduous leaf habit.

**Figure 3:**
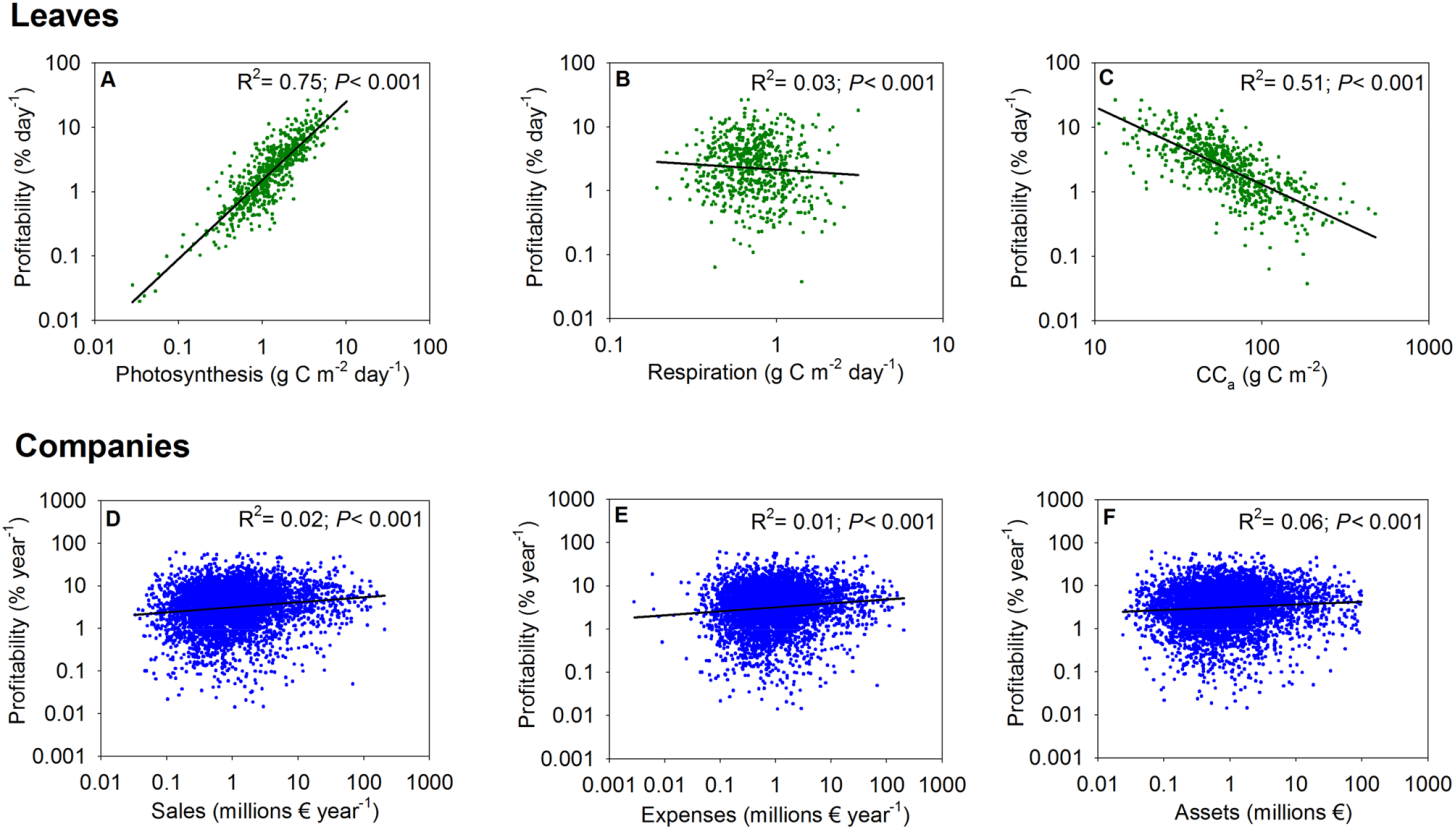
Factors related to leaf and company profitability. For leaves, relationships of profitability with (**A**) photosynthesis gains; (**B**) respiration expenses and (**C**)construction cost per leaf area (CC_a_). For companies, relationships of profitability with (**D**) sales; (**E**) expenses and (**F**) assets.

Finally, we want to know which factors can explain the differences in leaf economic profitability, for example if gains (photosynthesis), expenses (maintenance respiration) or assets (investment in construction) are related to profitability. Our results indicate that leaf profitability was strongly positively related to C-gain by photosynthesis (R^2^ = 0.75, *P* < 0.001; Fig. 3A) and negatively related to the leaf construction cost per leaf area (CC_a_, R^2^ = 0.51, *P* < 0.001; Fig. 3C).

Comparing leaves that developed in either low or high light, Poorter et al.^23^ found that the variable having strongest impact on payback time (the inverse of leaf profitability) was LMA, which is directly related to the construction cost per area^18^. Also, leaf profitability was negatively related to the C expenses due to maintenance respiration (R^2^ = 0.03, *P* < 0.001; Fig. 3B), but notably more weakly than were assets (CC_a_) or photosynthetic gains. Therefore, the predictions of Kikuzawa^15^ related to the effects of C gains and C costs on payback time (the inverse of profitability) are confirmed by our results.

The high variability in leaf profitability between different species leads us to ask why species with high leaf profitability are not more abundant. First, there is the question whether leaf profitability is related to whole-plant profitability. From the scarce data available (24 species, from^9,39^) we calculated the profitability of whole plants considering the expenses on construction and maintenance of stems and roots and found that it was lower (20 ± 7.3% day^−1^) compared to profitability of leaf (40 ± 11.4% day^−1^), but both profitabilities were strongly correlated (R^2^ = 0.74, *P* < 0.001, Supplementary File S5 online) and also positively correlated to relative growth rate (RGR) (R^2^ = 0.53, *P* < 0.0001 for leaf profitability; R^2^ = 0.83, *P* < 0.001 for plant profitability, Supplementary File S5 online). Therefore, we consider our values of leaf profitability to be a reliable indicator of profitability in a broad sense. One reason why there is a wide variation in leaf profitability may be that high profitability only is possible under certain conditions, which are very constrained in space or time—e.g. biomes with very short growth season (alpine, tundra); deciduous leaf strategies; etc. The high potential profitability of leaves from arctic and alpine regions may be seen not only as insurance for high probability of suboptimal conditions for the photosynthesis but also necessity for accumulation of storage for winter survival. Also, among co-occurring species, time-discounting^11^ equalises different LMA strategies when considered over the life of the investment. Other reasons could be the existence of trade-offs which may also imply that a high leaf profitability has some disadvantages as for example, leaves with high profitability (low LMA, high leaf N, etc.) tend to suffer higher rates of herbivory^36^ or operate at higher risk under drought^40^.

### Comparisons between leaf and company

Are the results of the leaf profitability analyses similar to those of companies? To answer this question, we conducted a similar analysis for a reference group of 4722 companies in Spain from ‘*Sistema de Análisis de Balances Ibéricos*’ (SABI)-database and we also use data of NASDAQ and Dow Jones companies to unravel the effect of different longevity. We calculated the economic profitability of companies and analysed the patterns in relation to company size, environment (type of sector) and durability, just as we did for leaves.

Mean values of company profitability were 5.3 ± 5.8% year^−1^ with a range of 0.41–15.5% year^−1^ (percentile 5 and 95%, respectively). It is noteworthy that the values of company profitability are much lower (average values of 5% year^−1^) than those for leaves (average values around 3.4% day^−1^) (please note that profitability for companies is by year, but for leaves is by day). The high values of leaf profitability can be due to the fact that they have to bear the costs of construction and maintenance of stems and roots^41^ and since the proportion of leaves respect to total biomass is usually low (around 5% under field conditions)^42^, leaves should be highly profitable to maintain the plant expenses.

We found a weak positive relationship between profitability and company size (measured by number of employees; Fig. 2D). Preliminary studies^43,44^ found that size (measured by assets or sales volume) had a positive influence on profitability, which they attributed to the market power of larger companies. However, later studies^45,46^ considered that intrinsic factors of the company predominantly explained the differences in profitability and that size is not relevant.

Whereas we analysed plant data with respect to biomes, in the case of companies we used sector type as a proxy for the environment, since it describes the market conditions in which companies must survive. As in the case of leaves, there were differences by economic sector (Fig. 2E), as previously reported by Schmalenesee^47^ and Wernerfelt and Montgomery^48^. The sectors of education, health, social and the scientific and technical sector had higher profitability (around 7% year^−1^), whereas the construction and primary industry sectors had the lowest. This difference is related to the role of innovation^49–52^ and the use of human capital^53–55^, and it is due to higher sales margins (they sell at higher prices). The innovator has a period of monopolistic gain before the imitators enter the market, and while they get established^56^.

It is difficult to find a mechanism to compare plants and companies in relation to longevity. Companies, unlike leaves, do not have a genetically predetermined longevity; therefore, they are not classifiable according to intrinsic factors such as life-span. Since technology companies have a shorter longevity^57^, we could draw a parallel between NASDAQ companies (technology companies) and deciduous leaves, and between Dow Jones companies and evergreens leaves, to establish a comparison. We calculated the relative growth rate of the stock market capitalization for both types of companies (Supplementary File S6 online). We found that NASDAQ companies, like deciduous leaves, showed a higher profitability (measured as stock market capitalization rate) than those of Dow Jones (Fig. 2F), as a consequence of the greater risk that the investor supports^58^.

Unlike the case of the leaves, company profitability was not well explained by any of the other variables considered: sales (R^2^ = 0.02, *P* < 0.001; Fig. 3D), expenses (R^2^ = 0.01, *P* < 0.001; Fig. 3E) or assets (R^2^ = 0.06, *P* < 0.01; Fig. 3F).

**Figure 4:**
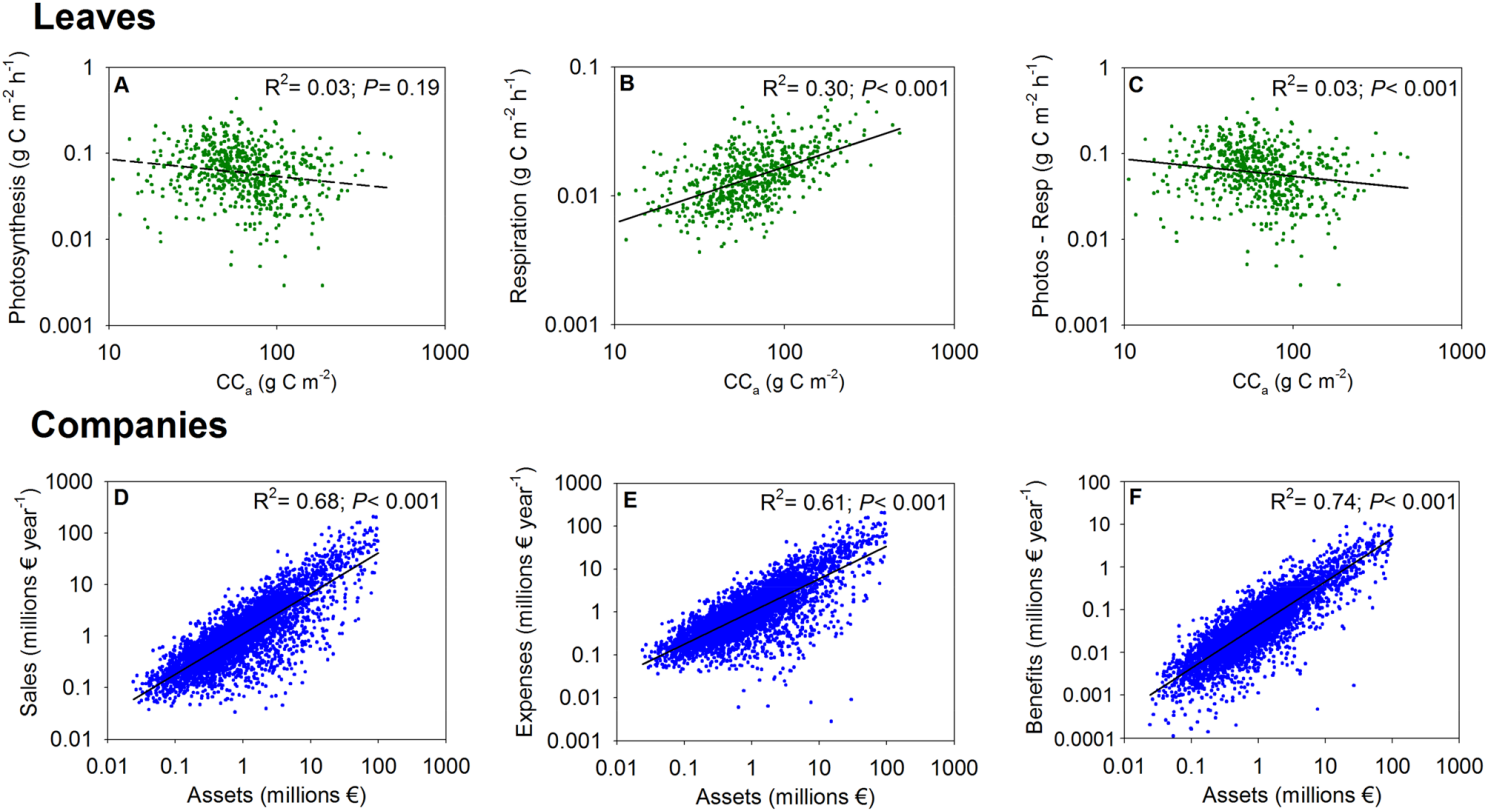
Relationships of (**A**) photosynthesis, (**B**) respiration and (**C**) benefits (photosynthesis-respiration) with construction cost per unit leaf area (CC_a_) for leaves. Relationships of (**D**) sales, (**E**) expenses and (**F**) benefits with assets for companies.

Statistically, the different results between leaves and companies can be understood because for leaves photosynthetic gains were unrelated to construction cost per leaf area (CC_a_) (Fig. 4A), whereas respiration expenses were positively related to CC_a_ (Fig. 4B), meaning that overall benefits (photosynthetic gains—respiratory expenses) were negatively related to CC_a_ (Fig. 4C). Thus, leaf profitability was negatively related to assets (CC_a_) (Fig. 3C). However, in the case of the companies there is a direct relationship between benefits and assets (with a slope of 1), since sales and expenses vary proportionally in similar amounts with the volume of assets (Figs. 4D–F). Therefore, the ratio of benefits/assets (profitability) became very constant and it is not well correlated with assets (R^2^ = 0.06, Fig. 3F).

The disparity between the results of the leaf economy and the companies is surprising a priori. However, from an economic point of view, this disparity can be understood as logical. In human economics, the resources migrate constantly from the less profitable companies to the more profitable ones^3^. Markets are in a permanent process of adjusting to equilibrium at a single rate of return through exchange, but they are also affected by a permanent innovative process of the companies that separate them from that equilibrium^56^. Conversely, it is not feasible for a plant to transfer part of its resources to another that is more productive (except for example for clonal plants). Leaf profitability depends, in addition to the available resources, on its genetically predetermined structure. There is no exchange and there is no adjustment towards a single rate of return. This difference may explain the contrasting results (Fig. 3A–C compared with Figs. 3D–F).

Boulding^59^ states that "*The world of economics is fundamentally organized by exchange*". This role of exchange, typical of the social facet of the human being, will always make a difference between plants and human beings. However, the plants are optimizing subjects and have economic behaviours in the sense of the Robbins definition. Therefore, identifying measurable variables of these processes, as we do in this work, broadens our perspective and may open unexplored paths in our study on issues in which the optimizing behaviour of plants is evident, e.g. accumulation of resources, growth, and limits to growth, etc. In the age of breaking of disciplinary limits^60^, we think that it is possible to extract advances in knowledge if we analyse the economic behaviour of plants.

We argue that the calculation and use of profitability at leaf and plant level can be a useful tool for understanding plant strategies, the success in certain environments and plant evolution. Profitability at the level of human economics is an essential and indisputable variable for understanding the economy and applying economic models. We have used the analogy between the company and the leaf and we have analysed similar trends that arise in relation to size, environment and durability. Therefore, the application of this variable in the context of plant functioning will facilitate conceptual advances and new understanding of plant evolution and ecological function.

## Supplementary Information

Supplementary Information.

## Data Availability

The datasets generated and analysed during the current study are available from the corresponding author on reasonable request.
