# Supplementary material for: Applying the economic concept of profitability to leaves
Source: Sci Rep. 2021 Jan 8;11:49. doi: 10.1038/s41598-020-79709-w (PMC7794281; doi:10.1038/s41598-020-79709-w)
Supplement: Supplementary file 1 — Supplementary Information. [file 41598_2020_79709_MOESM1_ESM.docx]

**Supplementary material**

**Crossing borders: applying the economic concept of profitability to plants**

**Rafael Villar, Manuel Olmo, Pedro Atienza, Antonio J. Garzón, Ian J. Wright, Hendrik Poorter, Luis A. Hierro**

**S1 File.** Explanation of the plant / company and leaf / company analogy

**S2 File.** Simple and complex calculations of leaf profitability.

**S3 File.** Calculations of Internal Rate of Return (IRR) or profitability.

**S4 File.** Comparison of results of leaf profitability following different approaches.

**S5 File.** Calculation of profitability for leaves, shoot and whole plant for 24 species grown under controlled conditions (Poorter and Remkes 1990).

**S6 File.** Calculation of the relative growth rate of stock market capitalization for both types of companies (Nasdaq and Dow Jones).

**S1 Table**. Economic and ecological definitions of terms used in this study.

**S1 File.** Explanation of the plant / company and leaf / company analogy.

The company can be analysed from multiple perspectives. If we adopt the conceptual approach to the economy as an exchange, the company is a market subject, competing with other companies, capturing productive factors (labour, capital and raw materials) to organize its production. From this perspective, which in essence is that adopted by Bloom et al (1985), the most appropriate analogy is that of plant / company. Thus, individual plants compete with others for the resources of the same zone. However, when we adopt the perspective of scarcity, companies present themselves as organizing entities of production. They use the available resources efficiently for production. Its objective is to obtain maximum production at minimum cost, maximizing profit and, consequently, the return on capital invested in it. From this perspective, the leaf / company analogy is as valid as the plant / company. The business concept is versatile enough to accommodate both analogies

To explain it we give an example. Suppose a national company that produces dairy desserts in a large country and to meet demand has invested in the construction of multiple factories (production plants) and in central offices for general administration activities. Each factory buys milk and other raw materials, hires workers, etc. produces desserts and sells them to consumers in their local area. The company's sales revenue is made up of the sum of the sales revenue of each factory and the company's costs are the sum of all the costs of all the factories plus the costs of central services. Obviously, we can calculate both the benefits of each factory and the benefits of the company, which will be the sum of the benefits of all the factories less the costs of central services.

From this perspective, a plant is perfectly comparable to this company, as Bloom et al. (1985) suggested, where the leaves can be compared with the factories of the company. The plant invests in the construction of the new leaves, each of which then produces carbohydrates (their sales) from light and CO_2_, consuming part of the carbohydrates to maintain their own function (maintenance costs) and transferring the rest of the carbohydrates to the plant (stem and roots) to allow their maintenance, growth, reproduction and survival. As in the case of the dairy company, we can calculate the benefits and profitability of each leaf (each factory), or the benefits of the factory as a whole (the plant). The benefits of the plant will be equal to the benefits of all of the leaves minus the costs of construction and maintenance of the stem and roots, as well as the construction of new leaves or reproductive structures.

In this example we see that the assimilation of concepts is of the type plant/company and leaf/factory. But is it feasible to consider leaves as companies rather than as factories? The answer to this question is yes. Let's imagine that the company that we have given as an example changes its configuration and decides to create a company for each factory and a holding company that owns all the companies to which the central services are assigned. Now each factory is a company and therefore the analogy leaf/company is appropriate.

The flexibility of the company concept is total. A company can be subdivided or integrated horizontally and vertically by mere business strategy and it is precisely this conceptual flexibility that makes feasible the leaf/company analogy that we maintain in this work and also the plant/company analogy proposed by Bloom et al. (1985). What is truly relevant for the purposes of the leaf/company analogy is that we can estimate the investment made, the production obtained and the cost of the factors incorporated into production. Any of the parts of the plant, leaves, stem or roots, can be analysed as a production process and therefore can be studied by analogy in comparison with companies. It is only necessary to have a currency unit that allows homogeneous evaluations at all scales. Furthermore, there are specific companies that can be considered very similar to certain parts of the plant. For example, the roots are very similar to extractive companies (mining, agricultural, fishing, ...) and the stems have great similarity to infrastructure and logistics companies.

**S2 File.** Simple and complex calculations of leaf profitability.

***Simple calculations of leaf profitability***

For the simple approach, leaf benefits were calculated as the difference between the maximum photosynthetic and respiration rates. To calculate leaf profitability (Internal Rate of Return, IRR) we estimated leaf benefits per day considering 12 hours of photosynthetic gain and 12 hours of respiration losses (note, photosynthesis here is net photosynthesis, that is, once daytime respiration costs have already been accounted for). Then, profitability was calculated as: 100 × [leaf benefits per day/ leaf construction cost]. Leaf construction cost per unit leaf area was calculated from Leaf Mass per Area, as explained in Methods. We calculate different values considering a variable reduction of photosynthesis and respiration with age: a) without reduction (no decreases of photosynthetic rates with leaf age), b) 1/2 or c) 2/3 reduction of maximum photosynthetic rates with leaf age). Complex calculations assume 1/2 reduction of maximum photosynthetic rates with leaf age and consider the length of favourable period (see methods).

These basic calculations were contrasted with other more complex calculations considering a decrease of 1/2 of photosynthesis and respiration rates with the leaf age (Kikuzawa 1991), and the duration of the photosynthetic period taking into account the favorable season (Kikuzawa et al. 2013) (see Methods and also below). These more complex calculations gave very similar conclusions to those described using the simplest ones (Extended Data Appendix 3).

***Complex calculations of leaf profitability. Estimation of favorable period, day length and daily hours of photosynthesis and respiration***

We defined the favorable period for each biome (Table S1) as being the set of consecutive months that satisfied the following conditions: (1) monthly mean 24-hour temperature ≥ 5 °C; and (2) monthly rainfall / evapotranspiration ≥ 0.05 (Kikuzawa et al 2013). To calculate monthly mean rainfall, temperature and evapotranspiration of each biome we selected different coordinates (at least 3) from the geographical range of each biome. Climatic data were obtained using the geovisualization tool for broadcasting climatic data *Global Climate Monitor* (<http://www.globalclimatemonitor.org>), which is based on the CRU TS3.21 version of the Climate Research Unit (University of East Anglia) database for 1951-2012. In general, favorable period decreases strongly with latitude and/or altitude, with values of around 90 days for Alpine biome to 364 days for Tropical rain forest (Table S1). The favorable and non-favorable period was calculated as the sum of days of favorable and non-favorable days, respectively. Mean annual rainfall and temperature calculated for each biome is shown in Fig. S1 and as we can see it matches the climatic conditions of the different biomes.

Day length (hours) for favorable and non-favorable period and each biome were calculated using the sunrise equation (Duffett-Smith and Zwart, 2011). The sunrise equation (Extended Eq. 4) was used to derive the time of sunrise and sunset for any solar declination and latitude:

cos(ω_0_) = -tan(φ) × tan(δ) (*Extended Eq. 4*)

where ω_0_ is the hour angle (degrees) at either sunrise (when negative value is taken) or sunset (when positive value is taken); φ is the latitude (in degrees) of the observer on the Earth; and δ is the solar declination.

Solar declination (δ, degrees) was calculated following the Extended Eq. 5:

δ = 23.45 × sin [360 × (284 + J)/ 365] (*Extended Eq. 5*)

J is the day of the year (1-365). The earth's equator is tilted 23.45˚ with respect to the plane of the Earth's orbit around the sun. At various times during the year, as the Earth orbits the sun, declination varies from 23.45˚ North/South. To synchronize the Sin curve with the calendar, the distance from the March equinox to the end of the year (284 days) are added.

Day length duration (hours day^-1^) was calculated following the Extended Eq. 6:

Day length duration = 2 × ω_0_ / 15 (*Extended Eq. 6*)

The sun moves across the sky at 15° per hour.

To calculate the daily hours of photosynthesis and respiration (hours day^-1^) for the favourable period we assumed that 4 hours per day (2 hour at dawn and 2 hours at sunset) the gain by photosynthesis was offset by the cost by respiration (Extended Eq. 7 and 8).

Daily hours of photosynthesis = day length duration – 4 (*Extended Eq. 7*)

Daily hours of respiration= 20 – daily hours of photosynthesis (*Extended Eq. 8*)

Daily hours of respiration for the non-favourable periods were calculated as: 24- day length in non-favourable periods.

**References**

Duffett-Smith P, Zwart J (2011) Practical Astronomy with your Calculator or Spreadsheet, 4th edn. Cambridg e University Press, Cambridge, UK.


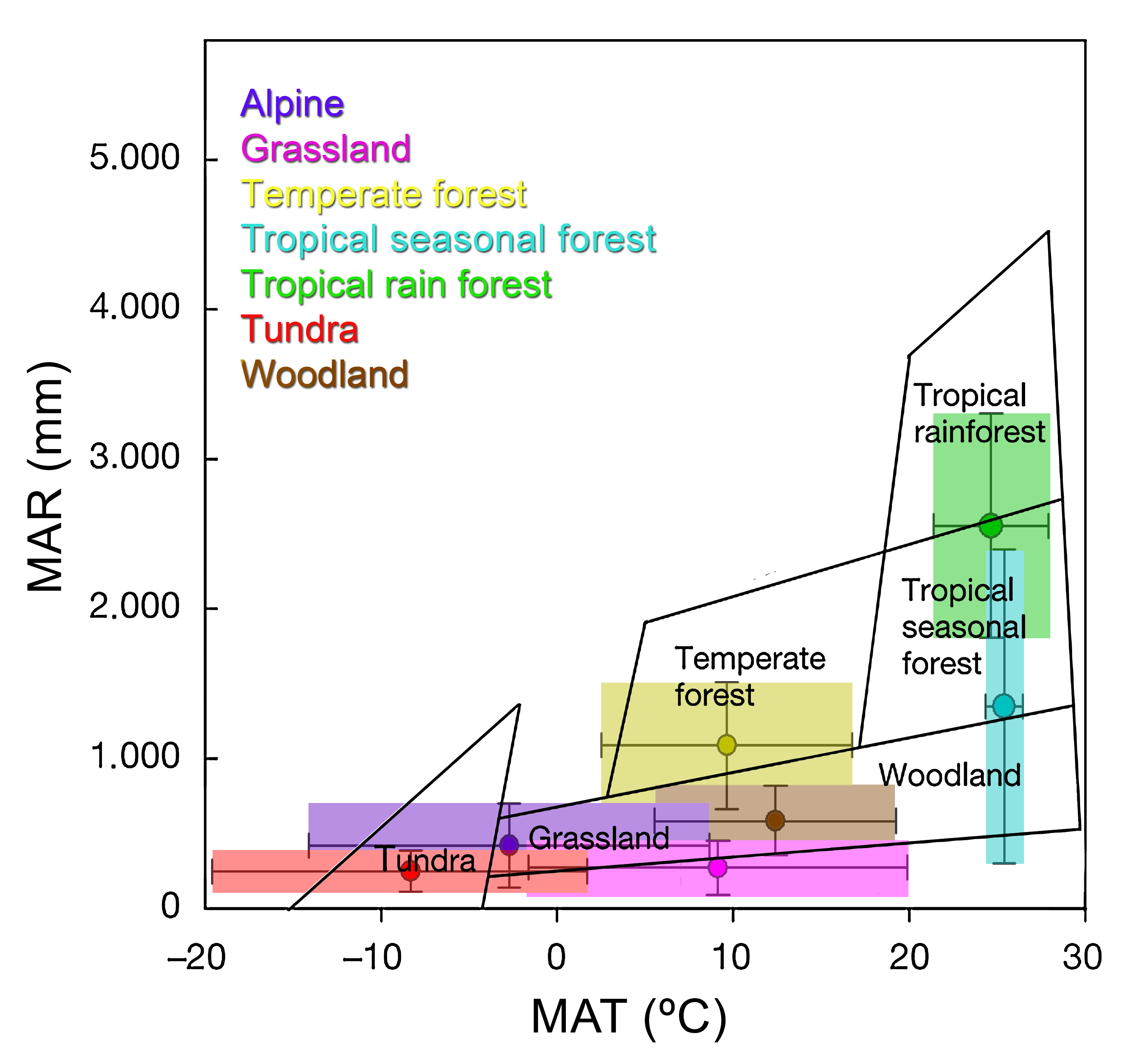


**Fig. S1 of S2 File.** Annual mean rainfall and temperature for each biome types of the world calculated following the approach described.

**Table S1 of S2 File.** Length of favorable period and day length, photosynthesis and respiration time of the biomes calculated following our approach.

| **Biome type** | **Favorable period**  **(months)** | | **Favorable period (day year^-1^)** | **Day length**  **(h day^-1^)** | | **Photosynthesis**  **(h day^-1^)** | **Respiration**  **(h day^-1^)** | |
| --- | --- | --- | --- | --- | --- | --- | --- | --- |
|  | **Northern hemisphere** | **Southern hemisphere** |  | **Favorable period** | **Non- Favorable period** | **Favorable period** | **Favorable period** | **Non- Favorable period** |
| Alpine | Jun-Aug | Dec-Feb | 90 | 14.65 | 11.09 | 10.65 | 9.35 | 12.91 |
| Grassland | Apr-Sept | Oct-Mar | 180 | 13.81 | 10.19 | 9.81 | 10.19 | 13.81 |
| Temperate forest | Apr-Oct | Sept-Mar | 210 | 13.75 | 9.6 | 9.75 | 10.25 | 14.4 |
| Tropical seasonal forest | Jan-Dec | Jan-Dec | 364 | 12 | 12 | 8 | 12 | 0 |
| Tropical rain forest | Jan-Dec | Jan-Dec | 364 | 12 | 12 | 8 | 12 | 0 |
| Tundra | Jun-Aug | Dec-Feb | 90 | 18.68 | 9.72 | 14.68 | 5.32 | 14.28 |
| Woodland | Feb-May and Sep-Dec | Mar-Jun and Ago-Nov | 240 | 11.44 | 13.06 | 7.44 | 12.56 | 10.94 |

**S3 File**. Calculations of Internal Rate of Return (IRR) or profitability.

Internal Rate of Return (IRR) of an investment is the discount rate that makes the updated value of the future net returns of an investment equal to the cost of disbursing the investment in the initial period. IRR is an estimate of the economic return implicit in an investment.

If we have an investment project that has a useful life of *n* years and an initial cost, *C_0_* at time 0, we can calculate the net return of each one of the years of useful life of the investment, Y_n_. Then,

*(Extended equation 1)*


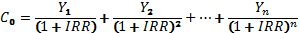

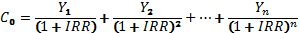


For constant net returns, *Y_1_=Y_2_=…=Y_n_=Y*,:

*(Extended equation 2)*


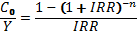

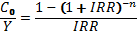


Extended equation 1 and 2 are nth degree equations that can have more than one solution and can be non-real solutions. However, when all *Y* are positive (or negative) there is only one positive and real solution and it can be calculated by approximation.

For perpetual investments with constant returns the calculation is more simple since the limit as n goes to infinity is:

*(Extended equation 3)*


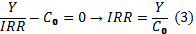

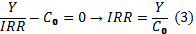


The IRR calculated in this way corresponds to the annual return measured as a percentage of the initial investment made C0. For example, an IRR = 10% means that the initial investment produced produces an annual return of 10% "

**S4 File**. Comparison of results of leaf profitability following different approaches.

In the complex calculations of leaf profitability we assume that photosynthetic and respiration rate decrease linearly with leaf age and the mean rate was around 50% of maximum rates. We analysed the importance of this assumption considering two scenarios: a) no reduction of the photosynthetic and respiration rate with leaf age, and b) a stronger reduction, with a value reaching only 30%. In Fig. S1 and S2 we can see that the leaf profitability decreases with the amount of reduction of photosynthetic and respiration rates with leaf age, but the different estimates are strongly correlated. Therefore, we conclude that these assumptions do not affect the main conclusions.


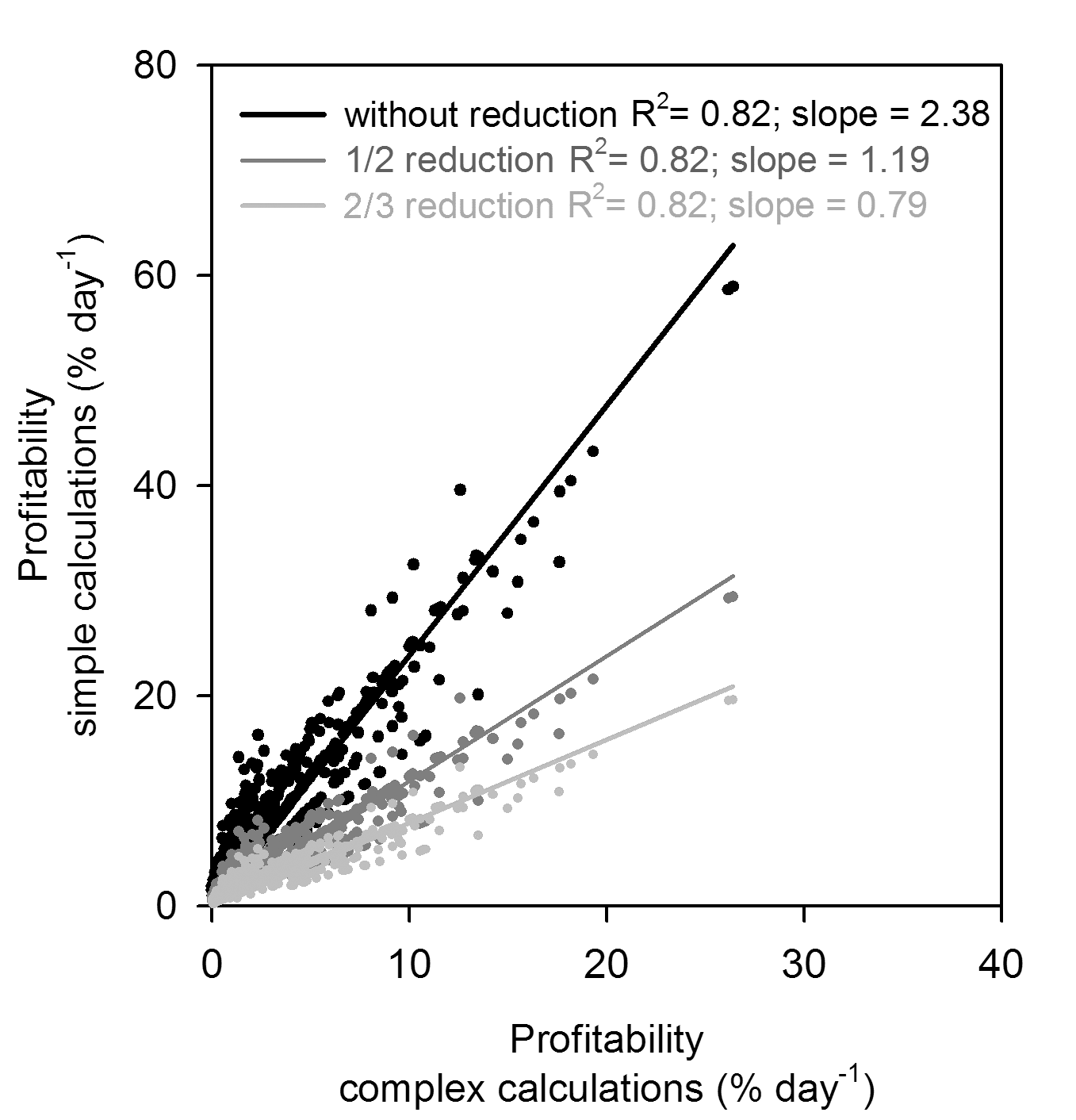


**Fig. S1 of S3 File.** Profitability of the leaf following different approaches: simple calculations versus complex calculations. Simple calculations: a) without reduction (no decreases of photosynthetic rates with leaf age), b) 1/2 or c) 2/3 reduction of maximum photosynthetic rates with leaf age). Complex calculations assume 1/2 reduction of maximum photosynthetic rates with leaf age and consider the length of favourable period (see methods).


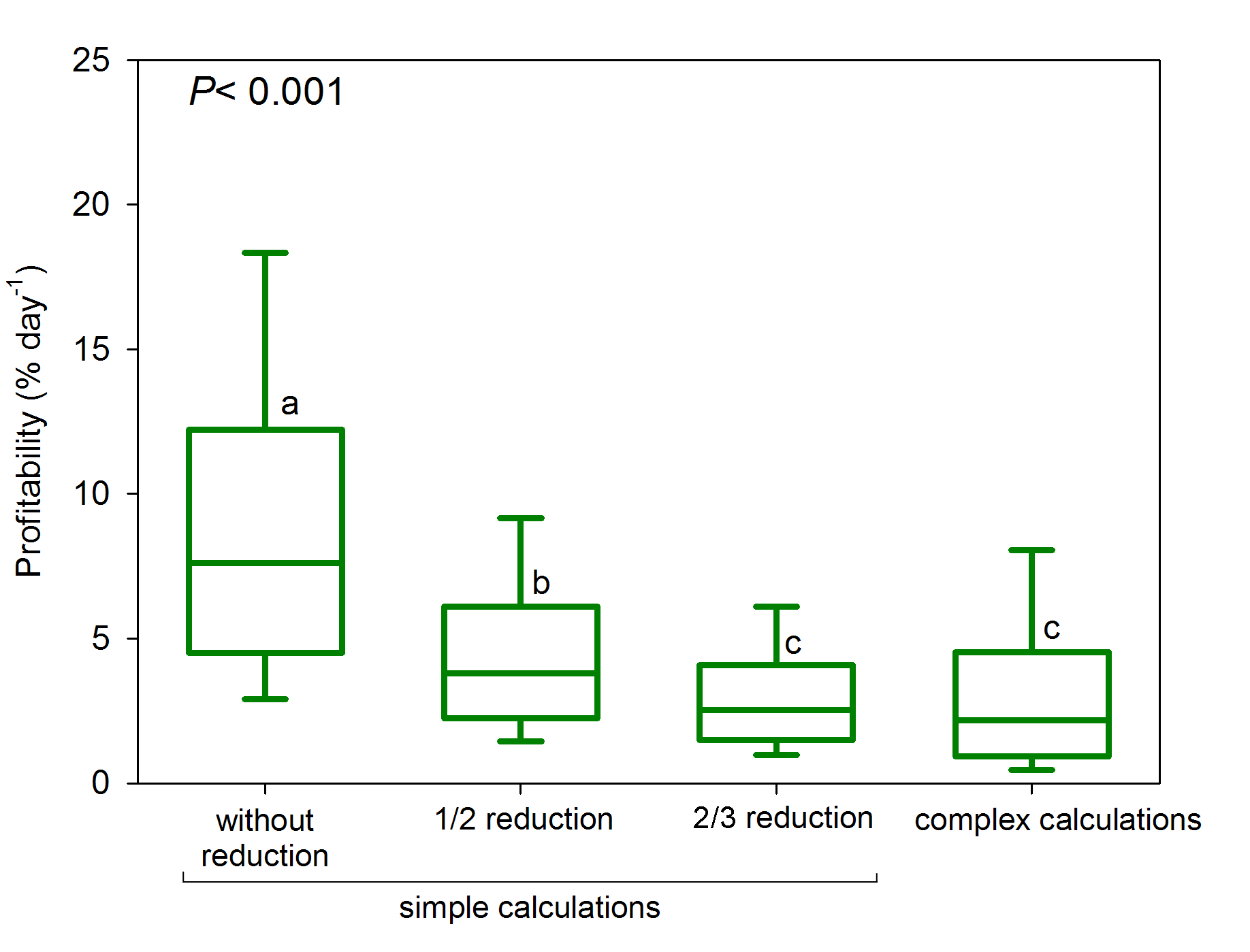


**Fig. S2 of S3 File.** Mean values of leaf profitability following different approaches: simple calculations versus complex calculations. Simple calculations: a) without reduction (no decreases of photosynthetic rates with leaf age), b) 1/2 or c) 1/3 reduction of maximum photosynthetic rates with leaf age). Complex calculations assume 1/2 reduction of maximum photosynthetic rates with leaf age and consider the length of favourable period (see methods). The box in each plot shows the median and the lower and upper quartile, and the whiskers show the range of variation.

**S5 File.** Calculation of profitability for leaves, shoot and whole plant for 24 species grown under controlled conditions (Poorter and Remkes 1990).

We used data of leaf photosynthetic and respiration rates, construction cost of leaf, stem and roots and the biomass allocation to leaf, stem and root to calculate the profitability of leaf, shoot and whole plants from the dataset in Poorter and Remkes (1990) and Poorter et al. (1990). The dataset is based in 24 herbaceous species growing under controlled conditions. Leaf profitability was calculated following the simple approach used in this paper (see methods and Extended Data Appendix 2). Profitability of shoot was calculated considering the amount of stem per unit total leaf area for each species, the expenses in construction and maintenance (respiration) of stem and leaf and the gains by photosynthesis. Profitability of whole plant was calculated considering the amount of stem and root per unit total leaf area for each species, the expenses in construction and maintenance (respiration) of stem, root and leaf and the gains by photosynthesis.

We found that the mean value of profitability decreases from around 40% day^-1^ for leaves to around 20% day^-1^ for whole plants (Fig. S1 Extended Data Appendix 4), but profitability for leaf, shoot and whole plants were strongly correlated (Fig. S2 Extended Data Appendix 4), indicating that the conclusions based on leaf profitability can stand for whole plants. We also found that profitability was strongly and positively correlated to relative growth rate (Fig. S3 Extended Data Appendix 4).

**Fig. S1 of S4 File.** Mean values of profitability of leaf, shoot and whole plants for 24 herbaceous species grown under controlled conditions (Poorter and Remkes 1990 and Poorter et al. 1990).


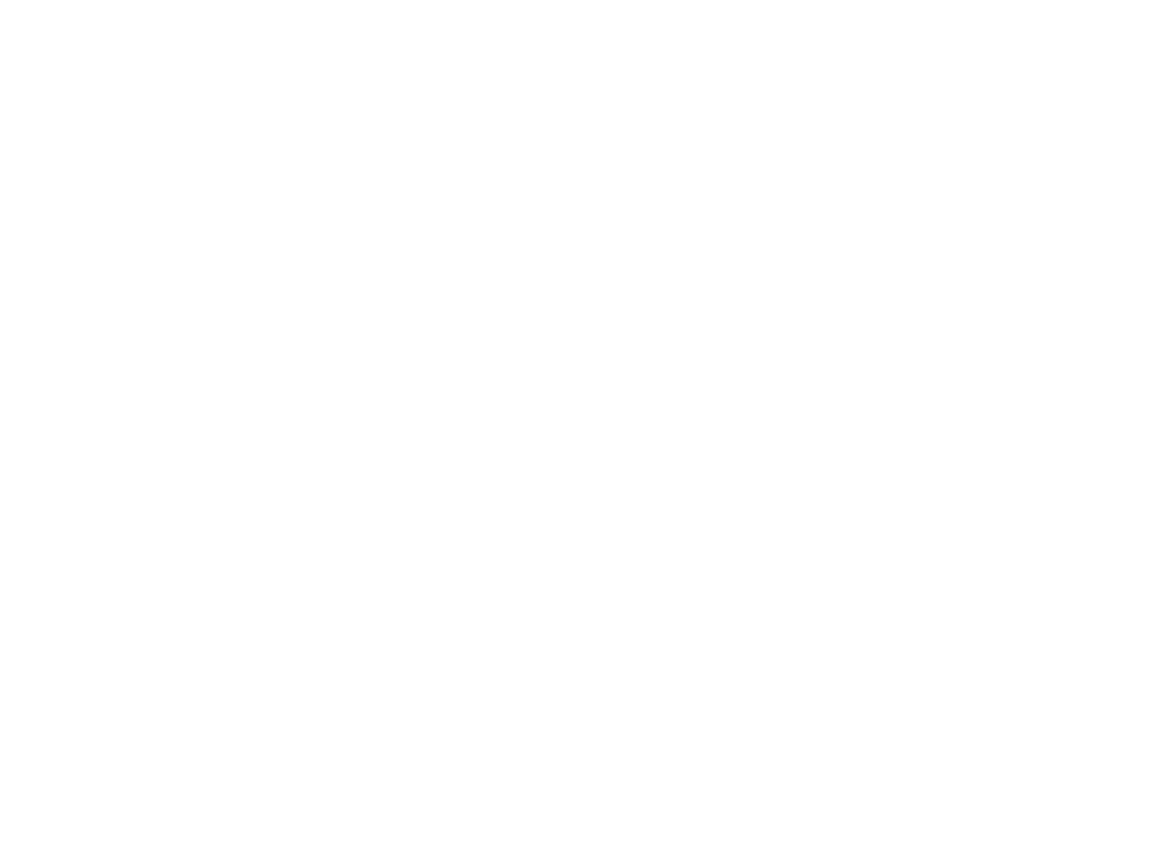

**Fig. S2 of S4 File.** Relationships between profitability of leaf with profitability of shoot and whole plant for 24 herbaceous species grown under controlled conditions (Poorter and Remkes 1990 and Poorter et al. 1990).

.


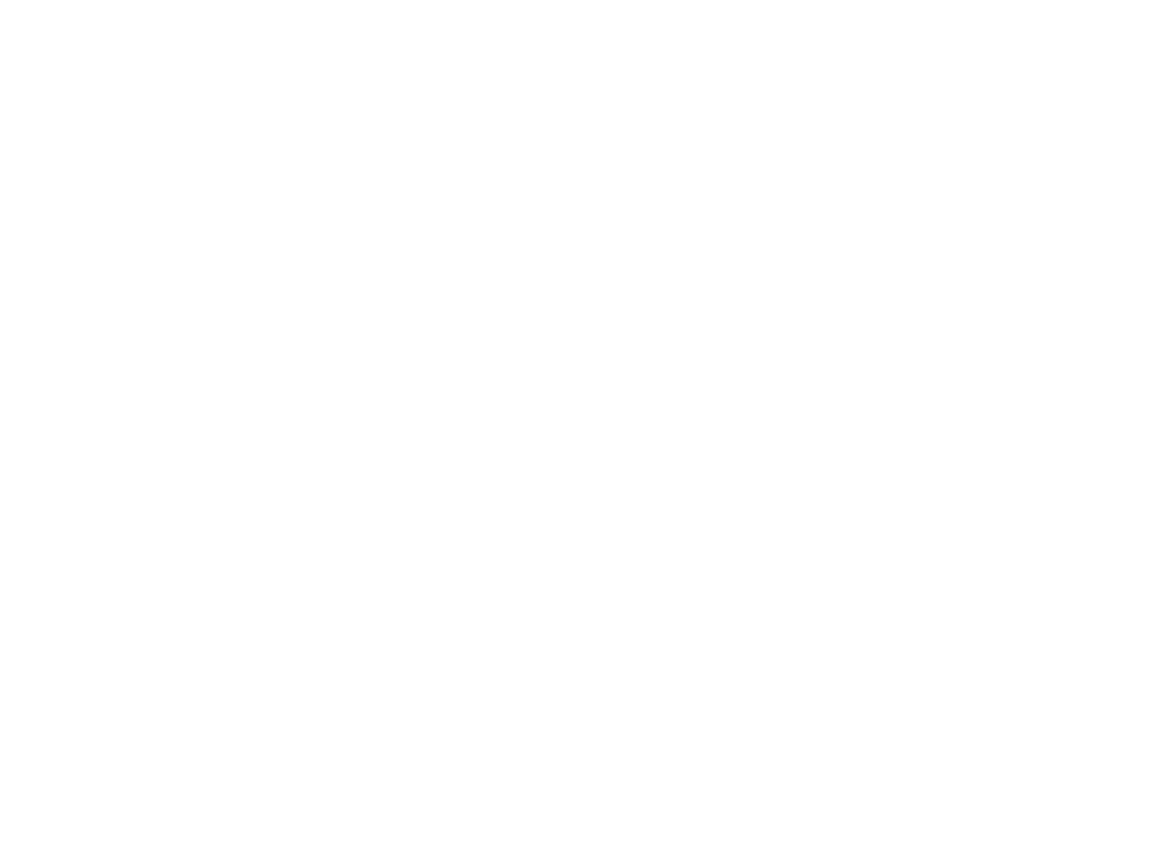

**Fig. S3 of S4 File.** Relationships between relative growth rate (RGR, mg g^-1^ day^-1^) with profitability of leaf shoot and whole plant for 24 herbaceous species grown under controlled conditions (Poorter and Remkes 1990 and Poorter et al. 1990).

**S6 File.** Calculation of the relative growth rate of stock market capitalization rate for both types of companies (NASDAQ and Dow Jones).

We have roughly estimated the profitability of the two types of companies by calculating the average growth rate of the value of each of the stock indices for the period 1971-2015 in real terms (i.e. by deducting the growth of prices every year). For that we log-transform the data and calculate the slope of the relationship of Log (stock indices) versus time, giving values of relative growth rate.


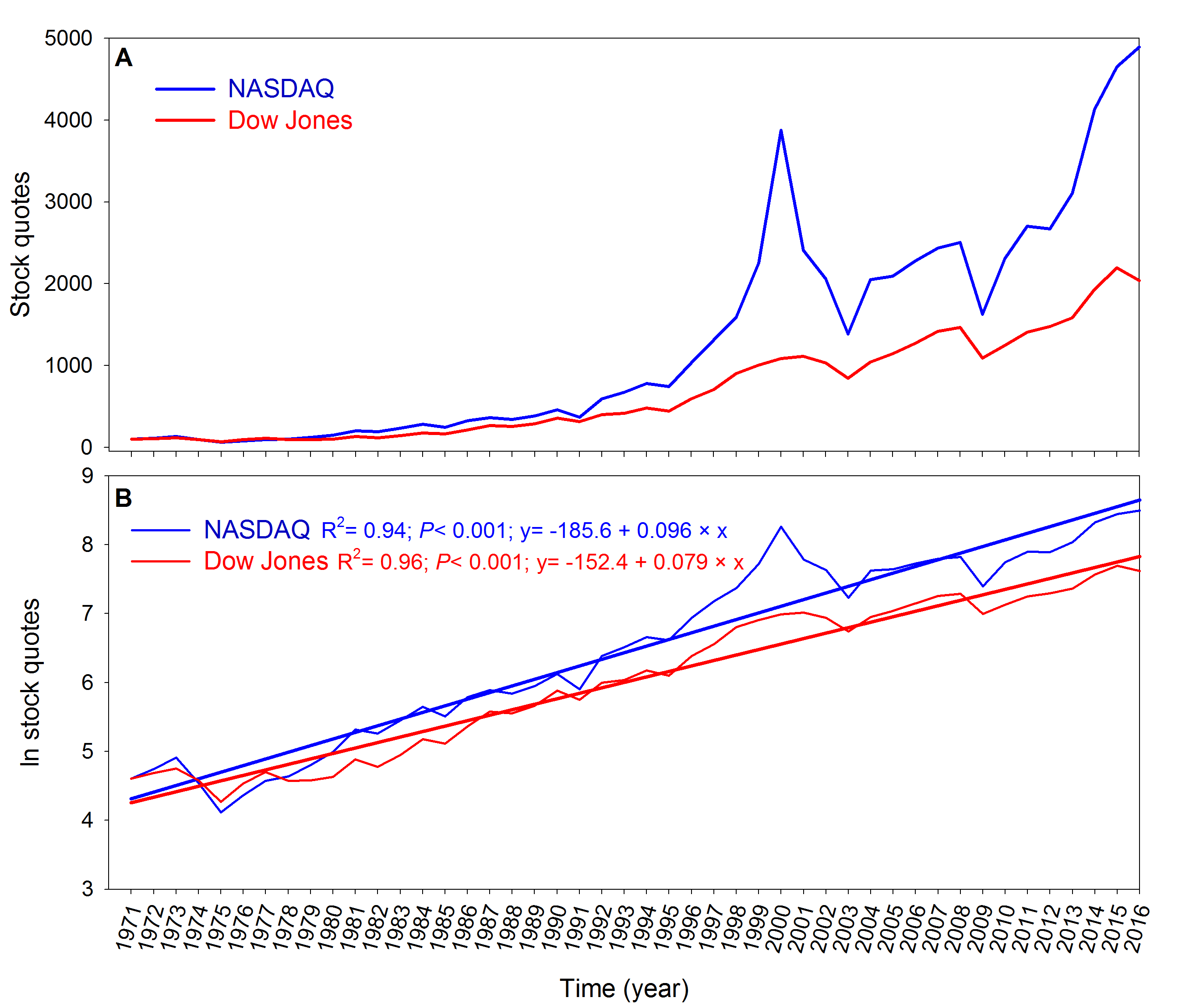


**Fig. S1 of S4 File. Time trends of stock indices for NASDAQ and Dow Jones companies.** (**A**) Temporal evolution of stock indices for NASDAQ and Dow Jones companies from 1971 to 2015. (**B**) Temporal evolution of Ln of stock indices for NASDAQ and Dow Jones to calculate the average growth rate as the slope of the relationship.

**S1 Table**. Ecological and economic definitions of terms used in this study.

| **Term** | **Economy** | **Ecology** |
| --- | --- | --- |
| *Functional unit of study* | *Company*  Commercial organization that operates on a for-profit basis and participates in selling goods or services to consumers | *Leaf*  Organ responsible for carbon exchange in plants |
| *Classification by environment* | *Economic sector*  The different categories of stocks that contain companies | *Biome*  Large ecological areas, defined by abiotic factors such as climate, relief, geology, soils; and by the vegetation that occurs there |
| *Gains* | *Sales revenue (€ year^-1^)*  The amount realized from selling goods or services in the normal operations of a company | *Net Photosynthesis (g C m^-2^ day^-1^)*  Process of net carbon gain by the leaf under light conditions |
| *Expenses* | *Expenses (€ year^-1^)*  Money spent for obtaining resources and for maintenance | *Respiration (g C m^-2^ day^-1^)*  Process of carbon losses by the leaf under darkness |
| *Profit* | *Profit (€ year^-1^)*  Sales revenue minus expenses before interest and tax | *Profit (g C m^-2^ day^-1^)*  Net profit of C obtained by subtracting respiratory C costs from the net photosynthetic C gains |
| *Construction cost* | *Assets (€)*  Tangible and intangible items that a company has acquired or purchased, and that have money value that derives its worth from its ability to be sold, used or bartered | *CC (g C m^-2^)*  Represents the investment in C required to construct a leaf |
| *Profitability* | *Profitability (%)*  Expresses the ability of the company to increase its assets. Also named *Internal rate of return (IRR)* | *Profitability (%)*  Percentage of investment in construction of the leaf obtained as surpluses and which can be used to manufacture other leaves or other plant parts |
